# Comparing SARS-CoV-2 neutralizing antibody levels in convalescent unvaccinated, convalescent vaccinated, and naive vaccinated subjects

**DOI:** 10.1016/j.heliyon.2023.e17410

**Published:** 2023-06-17

**Authors:** Latha Dulipsingh, Ernst J. Schaefer, Dorothy Wakefield, Kendra Williams, Adis Halilovic, Rebecca Crowell

**Affiliations:** a Saint Francis Hospital and Medical Center, 114 Woodland St., Hartford, CT, USA 06105; b Boston Heart Diagnostics, 200 Crossing Boulevard, Framingham, MA, USA, 01702

**Keywords:** SARS-CoV-2, Neutralizing antibodies, Convalescent unvaccinated, Convalescent vaccinated, Naive vaccinated

## Abstract

**Background:**

Severe acute respiratory syndrome coronavirus 2 (SARS-CoV-2) emerged in December 2019 and spread rapidly. The purpose of this study was to compare neutralizing antibodies (NAbs) following the original booster vaccine in convalescent and naive vaccinated individuals and in a third comparison group consisting of unvaccinated convalescent plasma donors.

**Methods:**

We assessed NAbs before and 2 months after a booster vaccine in 68 adults who had completed the initial vaccine series for SARS-CoV-2. Of these subjects, 58 had no history of prior infection (naïve vaccinated group) and 10 had been infected with SARS-COV-2 prior to the completing the first vaccine series (convalescent vaccinated group). A third comparison group included unvaccinated convalescent plasma donors (n = 55) from an earlier study with NAbs assessed approximately 2 months after a positive test for SARS-CoV-2.

**Results:**

Prior to the booster, convalescent vaccinated subjects had higher NAbs compared to naive vaccinated subjects (p = 0.02). Two months following the booster, NAbs increased in both vaccinated groups. The naive vaccinated group increased more than the convalescent vaccinated group (p = 0.02). NAbs in the naive vaccinated group were almost four times higher than NAbs in the 55 unvaccinated subjects, while the convalescent vaccinated group had levels 2.5 times higher p < 0.01.

**Conclusion:**

NAbs in both vaccinated/boosted groups were significantly higher than in the convalescent unvaccinated group (p < 0.01). Our data indicates that subjects with a single infection with SARS-CoV-2 did not have the same levels of neutralizing antibodies that we observed in subjects who were either in the convalescent vaccinated or the naive vaccinated groups.

## 1 Introduction

The severe acute respiratory syndrome coronavirus 2 (SARS-CoV-2) emerged in mid-December 2019 and spread rapidly. On March 11, 2020, the World Health Organization (WHO) declared this global spread a pandemic. SARS-CoV-2 is an enveloped positive-sense single-stranded RNA virus that ranges from 26 to 32 kb and is considered to possess the largest viral RNA genome. This large RNA covered by an envelope of nucleocapsid (N) protein held in place by a phospholipid bilayer and a complex of proteins including spike glycoprotein (S) and envelope (E) proteins resulting in a crown-like shape for SARS-CoV-2 [1]. Following SARS-CoV-2 infection, anti-viral antibodies are produced in response to the spike (S) and nucleocapsid (N) proteins and are commonly measured for serological testing [1]. The titer of IgM rises first as an initial T-independent humoral response to the virus entry and has a short half-life of 5–6 days. Then antigen presentation to the T cells leads to IgG production within a week and these antibodies tend to last much longer in serum.

It has been shown that implementation of safe and effective vaccine strategies will help prevent infection and hospitalizations [2,3]. The primary goal is to induce a sustained immune response to SARS-CoV-2. Vaccines developed initially against SARS-CoV-2, included the mRNA vaccines BNT162b2 (Pfizer/BioNTech) the mRNA-1273 (Moderna), and the Janssen COVID-19. All vaccines were found to be effective against the original strain of the virus. However, vaccine-induced immunity wanes over time and may be less responsive to variants [[2], [4], [5], [6]], indicating a need for additional booster vaccinations. The US Centers for Disease Control and Prevention (CDC) considers adults to be fully vaccinated once they have completed the initial vaccine series and received the most recent booster vaccine [3].

There has also been work around developing serologic tests which can be used either as diagnostic tools or to assess seroprevalence following infection or vaccination. Neutralizing antibodies (NAbs) mediate viral neutralization by inhibiting viral replication and blunting pro-inflammatory endogenous antibody response by binding the SARS-CoV-2 receptor-binding domain (RBD) of S glycoprotein. These antibodies are important for predicting effectiveness of convalescent plasma therapy [7,8] and to assess efficacy of vaccination [[9], [10], [11], [12]]. Cristiano et al. showed that monitoring NAb levels following vaccination is helpful in assessing degree of immunization and the protection status against reinfection or new infection with SARS-CoV-2 [13]. Similarly, results of the COVE trial suggest that NAbs could serve as surrogate markers for efficacy of mRNA vaccines against SARS-CoV-2 [11].

Following exposure to SARS-CoV-2 effective neutralization of the pathogen is mediated by IgG antibodies, but IgA and IgM antibodies also play a role in the process of neutralizing and clearance of pathogens and can last up to 10 months post infection [9]. Understanding the longevity of NAbs in natural versus vaccine induced immunity is especially important to the success of vaccination efforts and booster strategies. Research has focused on viral and antibody kinetics [14,15] and have addressed vaccine effectiveness following the primary vaccine series and the impact on the boosters. The purpose of this study was to compare Nabs values following the original booster vaccine in convalescent and naive vaccinated individuals. We also assessed data from a third comparison group consisting of unvaccinated convalescent plasma donors.

## 2 Materials and methods

### 2.1 Study participants

We assessed NAbs before and after a SARS-COV-2 booster vaccine in 68 adults who had completed the initial vaccine series for SARS-CoV-2. All subjects had received their second dose of BNT162b2 (Pfizer/BioNTech, n = 32) or mRNA-1273 (Moderna, n = 33) vaccines, or a single dose of Janssen-COVID-19 vaccine (n = 3) between January and May 2021. They received a booster vaccine upon study enrollment between October 21, 2021, and December 20, 2021. Neutralizing antibodies were measured 1 to 7 days before (baseline), and 56 ± 7 days after the booster vaccination. Of the 68 subjects, 58 had no history of prior infection (naive vaccinated). Ten subjects had been infected with SARS-CoV-2 prior to completing the first vaccine series (convalescent vaccinated). Blood samples at study enrollment were obtained on average 267 days after completion of the primary vaccine series. The booster dose was either the Pfizer/BioNTech (n = 27, 39.7%) or the Moderna mRNA (n = 41; 60.3%) vaccine and was not required to be homologous [16]. Eight subjects (80%) in the convalescent vaccinated group and 50 (86%) in the naive vaccinated group had homologous vaccines.

We also had data from an earlier study of 136 unvaccinated convalescent plasma donors that spanned April 2020 through early July 2020 [17]. Donors were assessed for SARS-CoV-2 using standard PCR testing until they were negative for the virus. Neutralizing antibodies were collected at each time point and used to confirm eligibility for plasma donation. During the study we obtained between one and five NAb measurements depending upon how long it took to obtain a negative test. For the purposes of analysis, we used data from this earlier study here to form a third study group (convalescent unvaccinated). The analysis allowed us to compare NAbs of naïve and convalescent vaccinated subjects with NAbs of unvaccinated individuals who had been infected with SARS-CoV-2 early in the pandemic. Neutralizing antibodies of convalescent plasma donors were not assessed until they enrolled in the study. Therefore, we used the date of the first positive (diagnostic) test as baseline and selected 55 convalescent plasma donors with NAb measurements 45 to 75 days (60 ± 15 days) after the first positive test for the convalescent unvaccinated group.

The research was approved by the Trinity Health Of New England Institutional Review Board (FWA 00020300, Approval # SFH-22-29). Men and women were included if they were ≥18–90 years of age, and all demographic and laboratory data were de-identified prior to analysis. Informed consent was obtained and the study was conducted according to established guidelines and regulations.

### 2.2 Neutralizing antibody chemiluminescence assay

The SARS-CoV-2 neutralizing antibody assay was obtained from Diazyme Laboratories (catalog number DZ901A, Poway, CA). This assay is a competitive chemiluminescence immunoassay based on the specific interaction between the SARS-CoV-2 spike protein RBD and the human angiotensin-converting enzyme 2 receptor (hACE2) on the surface of host cells. In the absence of SARS-CoV-2 neutralizing antibodies, hACE2 and RBD form complexes that generate a high chemiluminescent signal (measured in relative light units, RLU). In the presence of SARS-CoV-2 neutralizing antibodies originating from human serum or plasma, the interaction between hACE2 and RBD is compromised; and the chemiluminescent signal is reduced in a dose-dependent manner. The assay has been validated with a cell-based assay as previously described [18]. Serum samples (n = 33) with neutralizing antibody values ≥ 2.60 AU/mL all showed >98.0% inhibition of viral infection in cell-based assay validation studies. The assay was documented to have no interfering substances and to be specific for SARS-CoV-2 [10,19,20]. In our laboratory this assay was found to have within- and between-run coefficients of variation of <4.0%, with positive value being ≥0.90 AU/mL and a linear range up to 30 AU/mL [21,22]. This assay has received an FDA EUA.

### 2.3 Statistical analyses

Descriptive statistics were calculated for all data collected and compared by group. Demographic data for the three groups were extracted from the electronic health record system (EHR) and included gender (male, female), age in years, race (White, Black or African American, Asian and Unspecified), and Ethnicity (Hispanic, non-Hispanic). Continuous variables (NAbs, age, number of days between measurements) were compared using ANOVA; categorical variables (gender, age group, race, ethnicity) were compared using χ^2^ tests or Fisher's Exact test. Neutralizing antibody measurements were log10-transformed for all statistical analyses. Change in NAbs (pre-post) among the 2 vaccinated groups was assessed using a paired *t*-test. Using all 3 study groups, a multivariate linear regression examined the relationship between study group and NAbs at approximately 2 months (45 to 75 days) following the index date. Among the two vaccinated groups, index date was the date of the booster, and among the convalescent unvaccinated group, the index date was the date of the positive test. Covariates considered included gender, age group, race, ethnicity, and number of days between measurements. The final model chosen was based on smallest AIC. The Tukey-Kramer adjustment for multiple comparisons was used for post-hoc pairwise comparisons.

## 3 Results

### 3.1 Comparative analysis

The comparative analysis included 123 adults between the ages of 20 and 78. The naive vaccinated group included 58 subjects; the convalescent vaccinated group included 10 subjects. Both groups had NAbs measurements at baseline and approximately 2 months later (56 ± 7 days). The third group included the 55 convalescent unvaccinated subjects with NAbs data between 45 and 75 days of their positive test. Demographics for the three groups are shown in Table 1. As recorded in the EHR, subjects were predominantly White. There was no difference in average age or in the percent of patients older than 60 years. Subjects were primarily female; however, men were overrepresented in the convalescent unvaccinated group (p = 0.02).

**Table 1:** Demographics and Neutralizing Antibodies by vaccination status.

	Convalescent, Unvaccinated n = 55	Convalescent, Vaccinated n = 10	Naive, Vaccinated n = 58	*P*-Value
Gender, n (%)				0.02
Female	30 (54.5)	7 (70.0)	46 (79.3)	
Male	25 (45.5)	3 (30.0)	12 (20.7)	
Race, n (%)				0.94
White	46 (83.6)	8 (80.0)	49 (84.5)	
Non-White^a^	9 (16.4)	2 (20.0)	9 (15.5)	
Hispanic Ethnicity, n (%)	5 (9.3)	0 (0.0)	4 (6.9)	0.58
Age, mean (SD)	49.5 (13.9)	53.7 (15.4)	53.9 (14.0)	0.23
Age >60, n (%)	14 (25.5)	3 (30.0)	19 (32.8)	0.69
Neutralizing Antibody at baseline, mean (SD), AU/mL	n/a	3.4 (2.8)	1.7 (1.2)	0.02^c^
Neutralizing Antibody at endpoint, mean (SD), AU/mL	3.9 (4.2)	10.6 (4.2)	15.4 (8.7)^b^	<0.01^c^
Days between points^d^, mean (SD)	54.0 (7.7)	60.8 (4.6)	59.3 (4.0)^b^	<0.01

a Other race included: 9 Asian, 5 African American, and 6 unspecified.

b Four patients tested positive for COVID after the booster shot, so they are not included in the naive, vaccinated group at endpoint. They had a mean (SD) Nab 21.7 (11.2).

c Statistical comparisons used log10-transformed neutralizing antibody levels.

d Days between booster shot and date of antibody measurement for both vaccinated groups; days between positive test and date of antibody measurement for unvaccinated group.

Table 1 shows days between NAbs measurements. Overall, the interval averaged 57 days. The interval between positive test and final blood draw was significantly shorter in the convalescent unvaccinated donors compared to the interval from booster to final blood draw among naive and convalescent vaccinated subjects (p < 0.01). This was expected since the convalescent unvaccinated donor group was part of a different study and the difference between measurements was not pre-planned as in the current study.

Prior to the booster, convalescent vaccinated subjects had higher NAbs than naive vaccinated subjects (mean (SD): 3.4 AU/mL (2.85) vs 1.67AU/mL (1.25), p = 0.02, respectively). Four naive vaccinated subjects experienced a breakthrough infection between 2 and 58 days after the booster (mean = 25 days, SD = 26). None of the convalescent vaccinated subjects tested positive. Approximately 60 days following the booster, NAbs increased in both vaccinated groups. The naive vaccinated group increased more than the convalescent vaccinated group (Mean increase (SD): 13.7 AU/mL (8.7) vs 7.1 AU/mL (4.1), p = 0.02).

**Fig. 1:**
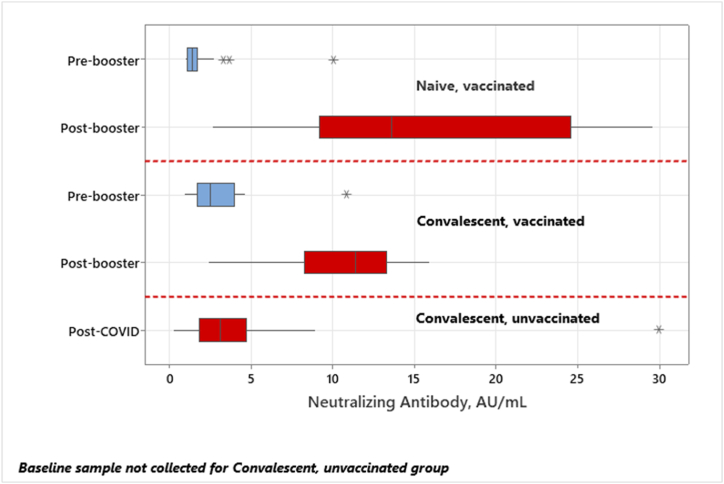
Figure Legend: Boxplots show distribution of Neutralizing Antibody levels (AU/mL) at baseline (pre-booster) and approximately 2 months later (post-booster).

Two months following the booster, both vaccinated groups had significantly higher NAbs compared to the convalescent unvaccinated donors (p < 0.01). NAbs in the naive vaccinated group were almost 4 times higher and NAbs in the convalescent vaccinated group and 2.5 times higher compared to NAbs in convalescent unvaccinated subjects (Table 1). There was no difference in the neutralizing antibodies post-booster between the naive vaccinated group compared to the convalescent vaccinated group (p = 0.46) naive (Fig. 1).

### 3.2 Regression analysis

Results of the multivariate regression predicting NAb concentration at 2 months among the 3 study groups are shown in Table 2. Covariates considered were patient gender, age group, race, ethnicity, and days between measurements. The best-fit model, with the smallest AIC (58.92), included study group, days between measurements, and gender. The final model included study group, days between measurements, and gender. Neutralizing antibodies among convalescent unvaccinated subjects (donors) were lower than NAbs among subjects in the vaccinated groups (p < 0.01). There was no difference in NAbs between naive vaccinated and convalescent vaccinated subjects (p = 0.63). Females tended to have higher NAbs than males (p = 0.04).

**Table 2:** Multivariate Regression predicting Neutralizing Antibody Level, AU/mL.^a^

Variables	Estimate (SE)	*P*-value
Study group		<0.01
Naive, vaccinated	0.69 (0.06)	
Convalescent, vaccinated	0.59 (0.06)	
Convalescent, unvaccinated (ref)	n/a	
Female	0.13 (0.06)	0.04
Number of days between measurement points^b^	−0.02 (0.004)	<0.01

a Regression used log10-transformed neutralizing antibody levels.

b Days between booster shot and date of antibody measurement for both vaccinated groups; days between positive test and date of antibody measurement for unvaccinated group. Four patients tested positive for SARS-COV-2 after the booster shot, so they are not included in the naive, vaccinated group at endpoint.

## 4 Discussion

Following infection or vaccination, antibodies targeting different epitopes of the virus or spike protein are produced, however not all effectively neutralize the virus and prevent binding of the RBD of the S protein to the hACE2 and prevent entry of SARS-CoV-2 into host cells. Neutralizing antibodies and cellular immunity are both important for protection against SARS-CoV-2 infection [11]. The gold standard for detection of NAbs is the plaque reduction neutralization test [23], however for this study we used a competitive chemiluminescence immunoassay based on the specific interaction between the SARS-CoV-2 spike protein RBD and the hACE2 on the surface of host cells. Binding assays tend to measure total antibodies, IgG, IgM and IgA.

Although the diagnostic value of serology testing for SARS-CoV-2 is limited, NAbs have shown to be useful in estimating immunity to natural disease and vaccinations within populations [12,24]. Presence of neutralizing antibodies post-infection is an essential component of antiviral immunity [11,25]. In our study NAbs were available for 55 unvaccinated convalescent plasma donors 45 to 75 days after their positive test, allowing comparison with convalescent and naive vaccinated subjects.

Vaccines produce NAbs which help reduce disease severity [26]. Hussain et al. [27] studied immune response and antibody titers in vaccinated inhabitants from different countries and found that vaccines inducing NAbs are best to reduce the SARS-CoV-2 ratio of a population. The antibody response following vaccination for SARS-CoV-2 may be more pronounced than the response induced by natural infection [[28], [29], [30], [31], [32]]. Trougakos et al. [15] and Saadat et al. [33] found that the strong immune response after one dose of booster vaccine may not suffice for naive recipients. In our study, NAbs in the convalescent vaccinated was higher than the naïve vaccinated pre-booster. Following the booster, the naïve vaccinated group had a larger increase so that at 2 months, there was no difference between the two vaccinated groups. With only 10 subjects in the convalescent vaccinated group, it is difficult to determine if this finding was random or resulted from the small sample size. In contrast, NAbs in the naive vaccinated group were almost 4 times higher than NAbs among the 55 unvaccinated donors, while convalescent vaccinated group had levels that were 2.5 times higher. Females had higher NAbs compared to males at 2 months. This finding is consistent with other studies, but the reason for the difference is unclear and warrants further study [30]. There was no association between age and NAbs, However, our sample size was small and older individuals were underrepresented.

Four naive vaccinated subjects developed SARS-CoV-2 following the booster compared to none of the convalescent vaccinated subjects. Vaccinated subjects completed the initial vaccine series between January 20, 2021, and May 16, 2021, just prior to emergence of the Delta variant in the United States in July of that year. Timing of our study coincided with the presence of the Delta variant and emergence of the Omicron B.1.1.529 variant and subvariants BA.2 and BA.2.12.1 [34]. Therefore, it is possible that the 4 naive vaccinated subjects were infected with one of these variants or subvariants. We have no way to confirm this, but if true this finding supports evidence that prior infection could provide an additional level of protection against infection by some variants and subvariants, including Delta and Omicron [6,2,35,36]. The booster vaccine has been shown to improve neutralization against the Delta and Beta variants [6,2,37]; it may be less effective against the Omicron variants, which evade immunity [37] and are the target of recent booster vaccines [36,38]. Unfortunately, testing for variants was outside the scope of this study.

Our study had several limitations. The sample size was small, and we did not assess comorbidities, severity of illness or other factors that may be associated with antibody levels, including body mass index, smoking status [30]. The small number of subjects receiving heterologous vaccinations precluded our ability to conduct a meaningful analysis based on homologous versus heterologous vaccines. We also did not test for SARS-CoV-2 variants that may have driven breakthrough infections seen in the naïve vaccinated group. Demographic data were limited to preexisting race and ethnicity categories within our electronic health record. Recent guidance by the American Medical Association (AMA) suggests that these categories are limiting, social constructs and that their use should be re-examined to provide meaningful information about health [39]. It is notable that our study population lacked diversity and included fewer individuals identified as non-White and fewer individuals who were older than 60. Including the convalescent unvaccinated subjects allowed us to compare the effect of natural immunity alone, but the subjects were from a different study, and we were confined to retrospective data. Subjects were convalescent plasma donors who were infected early in the pandemic and variants were not widespread. We also do not know if vaccinated subjects had an asymptomatic infection prior to or following the vaccine.

## 5 Conclusion

We found that at baseline, NAbs in the convalescent vaccinated was higher than the naïve vaccinated. Following the booster, the naïve vaccinated group had a larger increase in NAbs compared to the convalescent vaccinated group, so that at 2 months there was no difference between the two vaccinated groups. In contrast, at 2 months, NAbs in both vaccinated groups were 2.5 to 4 times higher than NAbs among the 55 unvaccinated donors. Our study supports findings from other studies [40] that a single infection with SARS-CoV-2 does not provide the levels of neutralizing antibodies as does the combination of either convalescent vaccinated or the naive vaccinated groups.

## Author contribution statement

Latha Dulipsingh, MD: Conceived and designed the experiments; Performed the experiments; Analyzed and interpreted the data; Wrote the paper. </p>

Ernst J Schaefer, MD: Conceived and designed the experiments; Analyzed and interpreted the data; Contributed reagents, materials, analysis tools or data. </p>

Dorothy Wakefield, MS; Adis Halilovic, BS; Rebecca Crowell, Ph.D: Analyzed and interpreted the data; Wrote the paper. </p>

Kendra Williams, BS: Performed the experiments; Analyzed and interpreted the data; Wrote the paper. </p>

## Data availability statement

Data will be made available on request.

## Declaration of competing interest

The authors declare that they have no known competing financial interests or personal relationships that could have appeared to influence the work reported in this paper..

## References

[1] Assadiasl S., Fatahi Y., Zavvar M., Nicknam M.H. (2020). COVID-19: significance of antibodies. Hum. Antibodies.

[2] Choi A., Koch M., Wu K., Chu L., Ma L., Hill A. (2021). Safety and immunogenicity of SARS-CoV-2 variant mRNA vaccine boosters in healthy adults: an interim analysis. Nat. Med..

[3] (2022). Prevention UcfDCa. Stay Up to Date with COVID-19 Vaccines Including Boosters.

[4] Evans D.J.R., Bay B.H., Wilson T.D., Smith C.F., Lachman N., Pawlina W. (2020). Going virtual to support anatomy education: a STOP GAP in the midst of the covid-19 pandemic. Anat. Sci. Educ..

[5] Chen X., Wang W., Wu Q., Sun R., Ge S., Zheng N. (2022). Prediction of long-term kinetics of vaccine-elicited neutralizing antibody and time-varying vaccine-specific efficacy against the SARS-CoV-2 Delta variant by clinical endpoint. BMC Med..

[6] Bellusci L., Grubbs G., Zahra F.T., Forgacs D., Golding H., Ross T.M. (2022). Antibody affinity and cross-variant neutralization of SARS-CoV-2 Omicron BA.1, BA.2 and BA.3 following third mRNA vaccination. Nat. Commun..

[7] Cao J., Wei J., Zhu H., Duan Y., Geng W., Hong X. (2020). A study of basic needs and psychological wellbeing of medical workers in the fever clinic of a tertiary general hospital in beijing during the COVID-19 outbreak. Psychother. Psychosom..

[8] Dou X., Wang E., Jiang R., Li M., Xiong D., Sun B. (2022). Longitudinal profile of neutralizing and binding antibodies in vaccinated and convalescent COVID-19 cohorts by chemiluminescent immunoassays. Immun. Inflamm. Dis..

[9] Montesinos I., Dahma H., Wolff F., Dauby N., Delaunoy S., Wuyts M. (2021). Neutralizing antibody responses following natural SARS-CoV-2 infection: dynamics and correlation with commercial serologic tests. J. Clin. Virol..

[10] Suhandynata R.T., Hoffman M.A., Kelner M.J., McLawhon R.W., Reed S.L., Fitzgerald R.L. (2020). Longitudinal monitoring of SARS-CoV-2 IgM and IgG seropositivity to detect COVID-19. J. Appl. Lab Med..

[11] Gilbert P.B., Montefiori D.C., McDermott A.B., Fong Y., Benkeser D., Deng W. (2022). Immune correlates analysis of the mRNA-1273 COVID-19 vaccine efficacy clinical trial. Science.

[12] Khoury D.S., Cromer D., Reynaldi A., Schlub T.E., Wheatley A.K., Juno J.A. (2021). Neutralizing antibody levels are highly predictive of immune protection from symptomatic SARS-CoV-2 infection. Nat. Med..

[13] Cristiano A., Nuccetelli M., Pieri M., Sarubbi S., Pelagalli M., Calugi G. (2021). Serological anti-SARS-CoV-2 neutralizing antibodies association to live virus neutralizing test titers in COVID-19 paucisymptomatic/symptomatic patients and vaccinated subjects. Int. Immunopharm..

[14] Zhang X., Lu S., Li H., Wang Y., Lu Z., Liu Z. (2020). Viral and antibody kinetics of COVID-19 patients with different disease severities in acute and convalescent phases: a 6-month follow-up study. Virol. Sin..

[15] Trougakos I.P., Terpos E., Zirou C., Sklirou A.D., Apostolakou F., Gumeni S. (2021). Comparative kinetics of SARS-CoV-2 anti-spike protein RBD IgGs and neutralizing antibodies in convalescent and naive recipients of the BNT162b2 mRNA vaccine versus COVID-19 patients. BMC Med..

[16] Atmar R.L., Lyke K.E., Deming M.E., Jackson L.A., Branche A.R., El Sahly H.M. (2022). Homologous and heterologous covid-19 booster vaccinations. N. Engl. J. Med..

[17] Dulipsingh L., Ibrahim D., Schaefer E.J., Crowell R., Diffenderfer M.R., Williams K. (2020). SARS-CoV-2 serology and virology trends in donors and recipients of convalescent plasma. Transfus. Apher. Sci..

[18] Muruato A.E., Fontes-Garfias C.R., Ren P., Garcia-Blanco M.A., Menachery V.D., Xie X. (2020). A high-throughput neutralizing antibody assay for COVID-19 diagnosis and vaccine evaluation. Nat. Commun..

[19] Suhandynata R.T., Hoffman M.A., Kelner M.J., McLawhon R.W., Reed S.L., Fitzgerald R.L. (2020). Multi-platform comparison of SARS-CoV-2 serology assays for the detection of COVID-19. J. Appl. Lab Med..

[20] Suhandynata R.T., Bevins N.J., Tran J.T., Huang D., Hoffman M.A., Lund K. (2021). SARS-CoV-2 serology status detected by commercialized platforms distinguishes previous infection and vaccination adaptive immune responses. J. Appl. Lab Med..

[21] Schaefer E.J., Dulipsingh L., Comite F., Jimison J., Grajower M.M., Lebowitz N.E. (2021 Jun 10). Corona Virus Disease-19 serology, inflammatory markers, hospitalizations, case finding, and aging. PLoS One.

[22] Dulipsingh L., Lang M., Diffenderfer M.R., Cook L., Puff J., Diaz L., He L., Schaefer E.J. (2023 Feb). Severe acute respiratory syndrome corona virus-2 (SARS-CoV-2) serology in the vaccination era and post booster vaccination. J. Clin. Virol..

[23] Gillot C., Favresse J., Maloteau V., Dogné J.M., Douxfils J. (2021). Dynamics of neutralizing antibody responses following natural SARS-CoV-2 infection and correlation with commercial serologic tests. A reappraisal and indirect comparison with vaccinated subjects. Viruses.

[24] European Centre for Disease Prevention and Control (2022).

[25] Wheeler S.E., Shurin G.V., Yost M., Anderson A., Pinto L., Wells A. (2021). Differential antibody response to mRNA COVID-19 vaccines in healthy subjects. Microbiol. Spectr..

[26] Garcia-Beltran W.F., Lam E.C., Astudillo M.G., Yang D., Miller T.E., Feldman J. (2021). COVID-19-neutralizing antibodies predict disease severity and survival. Cell.

[27] Hussain A., Rafeeq H., Asif H.M., Shabbir S., Bilal M., Mulla S.I. (2021). Current scenario of COVID-19 vaccinations and immune response along with antibody titer in vaccinated inhabitants of different countries. Int. Immunopharm..

[28] Forgacs D., Jang H., Abreu R.B., Hanley H.B., Gattiker J.L., Jefferson A.M. (2021). SARS-CoV-2 mRNA vaccines elicit different responses in immunologically naive and pre-immune humans. Front. Immunol..

[29] Anichini G., Terrosi C., Gandolfo C., Gori Savellini G., Fabrizi S., Miceli G.B. (2021). SARS-CoV-2 antibody response in persons with past natural infection. N. Engl. J. Med..

[30] Levin E.G., Lustig Y., Cohen C., Fluss R., Indenbaum V., Amit S. (2021). Waning immune humoral response to BNT162b2 covid-19 vaccine over 6 months. N. Engl. J. Med..

[31] Favresse J., Gillot C., Di Chiaro L., Eucher C., Elsen M., Van Eeckhoudt S. (2021). Neutralizing antibodies in COVID-19 patients and vaccine recipients after two doses of BNT162b2. Viruses.

[32] Zhang S., Xu K., Li C., Zhou L., Kong X., Peng J. (2022). Long-Term kinetics of SARS-CoV-2 antibodies and impact of inactivated vaccine on SARS-CoV-2 antibodies based on a COVID-19 patients cohort. Front. Immunol..

[33] Saadat S., Rikhtegaran Tehrani Z., Logue J., Newman M., Frieman M.B., Harris A.D. (2021). Binding and neutralization antibody titers after a single vaccine dose in health care workers previously infected with SARS-CoV-2. JAMA.

[34] CDC museum COVID-19 timeline. https://www.cdc.gov/museum/timeline/.

[35] Evans J.P., Zeng C., Carlin C., Lozanski G., Saif L.J., Oltz E.M. (2022). Neutralizing antibody responses elicited by SARS-CoV-2 mRNA vaccination wane over time and are boosted by breakthrough infection. Sci. Transl. Med..

[36] Lavezzo E., Pacenti M., Manuto L. (2022). Neutralising reactivity against SARS-CoV-2 Delta and Omicron variants by vaccination and infection history. Genome Med..

[37] Pulliam J.R.C., van Schalkwyk C., Govender N., von Gottberg A., Cohen C., Groome M.J. (2022). Increased risk of SARS-CoV-2 reinfection associated with emergence of Omicron in South Africa. Science.

[38] (2022). CDC Recommends the First Updated COVID-19 Booster [Internet].

[39] Flanagin A., Frey T., Christiansen S.L. (2021 Aug 17). AMA manual of style committee. Updated guidance on the reporting of race and ethnicity in medical and science journals. JAMA.

[40] Wratil P.R., Stern M., Priller A., Willmann A., Almanzar G., Vogel E. (2022). Three exposures to the spike protein of SARS-CoV-2 by either infection or vaccination elicit superior neutralizing immunity to all variants of concern. Nat. Med..

